# Self-Injury with Carbamazepine Intoxication in an Elementary School-Aged Child

**DOI:** 10.1155/2022/5135456

**Published:** 2022-05-30

**Authors:** Andrew Shieh, Natalie Schellpfeffer

**Affiliations:** ^1^Department of Emergency Medicine, University of Michigan, Ann Arbor, MI, USA; ^2^Department of Pediatrics, University of Michigan, Ann Arbor, MI, USA

## Abstract

Carbamazepine is a common anticonvulsant medication used to treat seizure disorders and is generally considered a safe medication. We describe the case of a 9-year-old female who presented with acute altered mental status and respiratory failure requiring mechanical ventilation. She was found to be intoxicated with carbamazepine through a urine drug test which was confirmed by bloodwork. After her medical condition improved, the patient admitted to self-injury through ingestion to cope with the death of a family member. She received a complete psychiatric assessment and was eventually discharged without permanent neurologic sequelae. To our knowledge, this is the first case of intentional self-injury with carbamazepine intoxication in an elementary school-aged child. When intoxication is suspected in children presenting with altered mental status, all medications available at home should be investigated. Preadolescent children may engage in nonfatal self-injury behavior, and diagnosis requires a high index of suspicion.

## 1. Introduction

Nonsuicidal self-injury is behavior in which a person intentionally inflicts injury on oneself in a way that is impulsive without the intent to commit suicide, and unfortunately, is common among adolescents [1–3]. Self-injury may include cutting, head banging or hitting, burning, or ingestion [1–3]. The behavior appears to increase as a child progresses through puberty, with some studies suggesting higher rates of self-injury in college-aged children [4–6]. Potential factors contributing to this increase include increased stress, alcohol consumption, and greater availability of medications [2, 4]. However, the exact prevalence of nonsuicidal self-injury in preadolescent children is not known. There are few studies that have highlighted the presence of both passive and active suicidal ideation in young children, and self-injury is thought to be rare in preteen children [6]. Furthermore, intoxication with medications at time of self-injury in elementary school-aged children has not been well described and is worthy of attention, as prior studies have identified only cutting, burning, self-hitting, and skin pricking as behavioral methods within this age group [6]. We present a case of intentional ingestion of carbamazepine in an elementary school-aged child to discuss the management of carbamazepine poisoning and highlight the capability of young children to commit self-injury.

## 2. Case Presentation

A previously healthy 9-year-old girl presented to the emergency department with altered mental status. She had intermittent episodes of tremor and screaming while sleeping at home four hours prior to arrival. Her parent went to her room to wake her up in the morning but instead, found her unarousable and unresponsive. Her parent denied known ingestion of any medications. There was no family history of psychiatric disorders or suicide. An older relative lived at home who was prescribed medications for epilepsy, but the patient's parent stated all medications were out of the patient's reach. The patient attended third grade in elementary school.

On physical examination, the patient's vital signs revealed a temperature of 35°C, heart rate of 107 beats/minute, respiratory rate of 28 breaths/minute, and a blood pressure of 109 mmHg/63 mmHg. The patient's oxygen saturation was 97% on room air. The patient was noted to be obtunded. Her eyes were closed with the right pupil measuring 4 mm in size and left pupil 2 mm in size, both sluggishly reactive to light. Neurologically, the patient was noted to have no spontaneous movement, increased tone, and no facial asymmetry. She was not responsive to painful stimuli. Her deep tendon reflexes were normal. The patient did not have tremors or lower extremity clonus. Sensation and strength could not be formally assessed.

In her laboratory findings, her complete blood cell count was unremarkable. Her electrolyte panel was notable for glucose of 181 (normal 50–135 mg/dL). Her serum acetaminophen, ethanol, and salicylate levels were undetectable. Her thyroid-stimulating hormone level was normal. The venous blood gas analysis showed a pH of 7.18 (normal 7.32–7.43), pCO_2_ 65 mmHg (38–50 mmHg), and HCO_3_ of 24 (normal 18–28 mmol/L). Her lactate was 5.7 (normal 0.5–2.2 mmol/L). Electrocardiogram revealed normal sinus rhythm. A computed tomography scan of the head revealed no acute intracranial abnormalities.

A dose of intravenous naloxone was given without improvement in her mental status, and she remained lethargic. The patient received rapid-sequence induction of anesthesia and was emergently intubated for airway protection due to acute hypercapnic respiratory failure. She was placed on a cardiac monitor, given intravenous fluids, started on continuous intravenous midazolam and fentanyl infusions, and admitted to the pediatric intensive care unit. She was initially treated empirically with ceftriaxone and vancomycin for concern for encephalitis. A lumbar puncture was not performed given the patient's gas chromatography screen of urine returned detecting carbamazepine prior to the procedure. A serum carbamazepine level was subsequently obtained and was found to be elevated at 30.2 *μ*g/mL, suggestive of carbamazepine overdose.

During her stay in the intensive care unit, her blood culture remained negative, and antibiotics were stopped after two days. Electroencephalographic monitoring revealed diffuse background slowing and disorganization with rudimentary sleep architecture indicating mild to moderate encephalopathy. Magnetic resonance imaging of her head revealed no intracranial abnormalities. After the urine results were confirmed, her parent revealed that the relative at home had disclosed an unknown number of carbamazepine pills were missing. Serial serum carbamazepine levels were obtained, which began to decrease on day one of hospitalization (Table 1). The patient was extubated and had no seizures.

Her neurological exam and mental status improved as her carbamazepine level continued to decrease, although she was noted to have dysarthria until hospital day four. The patient admitted she had taken the medications on her own free will to cope with the loss of a recent family member, but stated she was not intentionally trying to harm herself or commit suicide. She was unable to divulge why she could not explain her feelings to her caregiver and could not provide the exact time or dosage of ingestion. Her parent was unaware her daughter was overwhelmed with complicated grief. The patient did not disclose suicidal intent and denied she was being bullied at school. Psychiatry service was consulted and recommended outpatient resources for grief counseling and educated her parent to be vigilant about the location of all medications and weapons in the house. Prior to discharge, the patient had another electroencephalogram which revealed resolution of right temporal spikes and improvement of right posterior temporal polymorphic delta slowing. The incident was reported to child protective services who facilitated the patient to be discharged home safely after one week in the hospital. She was not prescribed any antiepileptic medications.

## 3. Discussion

Carbamazepine, known by its brand name Tegretol, is a tricyclic compound with mood-stabilizing and anticonvulsant properties [7, 8]. Today, the medication is particularly effective in the management of partial seizures, generalized epilepsy, migraines, postherpetic neuralgia, and manic-depressive disorders [8]. Patients who are prescribed carbamazepine for therapy require routine monitoring of blood levels since high levels may cause side effects including drowsiness, motor impairment, ataxia, and vomiting [7]. The recommended therapeutic level of carbamazepine in the blood is 4–12 *μ*g/mL [8]. In massive carbamazepine overdose, symptoms include headache, tremor, rigidity, diplopia, dysarthria, nystagmus, seizures, and coma [7–9]. The medication exerts anticholinergic effects on the stomach by decreasing motility and promoting absorption through enterohepatic recycling. Carbamazepine metabolites can be detected by urine drug screening with immunoassay and gas chromatography-mass spectrometry. In children, serum carbamazepine levels between 27 and 35 *μ*g/mL are considered the upper limit of severe toxicity, and higher levels are usually fatal [7].

The acute management of carbamazepine intoxication is similar to other cases of toxic ingestion and is primarily supportive with emergent attention to airway management and seizure control. Children should be emergently intubated and receive mechanical ventilation if comatose or if the child has inadequate respirations with severe hypercapnia on blood gas analysis. Patients should be placed on a cardiac monitor to assess vital signs and identify arrhythmias. Activated oral charcoal with a cathartic is recommended if there are no contraindications and should be repeated every two to six hours [7]. Clinicians should exercise careful judgment, as patients are frequently obtunded with suppressed pharyngeal reflexes and seizures, both of which pose a high risk for aspiration. Seizures generally respond to benzodiazepines, and if refractory, phenobarbital is recommended [7, 8]. Phenytoin should be avoided as it has a similar mechanism of action as carbamazepine and is unlikely to produce clinical benefit. The sodium channel blockade of carbamazepine and phenytoin may also increase risk of ventricular arrhythmias if used together [8]. There is currently no evidence that administration of sodium bicarbonate to alkalinize the serum is effective to treat carbamazepine intoxication, unlike intoxications with other medications that block sodium channels. If control of seizures cannot be achieved, a continuous infusion of midazolam or pentobarbital may be necessary [8]. Hypotension may require intravenous fluids and support with vasopressor medications. In young adults with convulsions, respiratory depression, coma, and levels greater than 37 *μ*g/mL, hemodialysis has been shown to effectively lower carbamazepine levels but not length of stay [9]. Hemodialysis is not proven to be efficient in removing carbamazepine after overdose in children.

Accidental or intentional ingestion of medications, whether belonging to the patient or other people in the home, should always be considered in children who present with altered mental status. After medical stabilization, patients should be evaluated for self-injurious behavior. Self-injury is common among children and poses a major public health burden because it is a strong predictor of suicide [10]. The term “self-injury” encompasses any deliberate, nonfatal behavior to purposefully damage one's own body, regardless of the presence of suicidal ideation or intent [10]. In 2013, “nonsuicidal self-injury” was included in the Diagnostic and Statistical Manual of Mental Disorders and requires six criteria to meet diagnosis including intentional self-inflicted damage on five or more days; to obtain relief from a negative feeling, cognitive state, or interpersonal difficulty; association with interpersonal difficulties or negative feelings or thoughts; not socially sanctioned behavior; behavior causes significant distress or interference in important areas of functioning; and behavior does not occur exclusively during psychotic episodes, delirium, substance intoxication, or substance withdrawal [11]. Self-injury encompasses directly dangerous behaviors including, and not limited to cutting or burning, as well as other activities including ingesting poisons and interfering with wound healing [2, 3].

There are few studies that have evaluated the prevalence and natural history of self-injury in elementary school-aged children. This is a particularly important period of a child's life, as studies have shown a rise in deaths from self-injury during this time [3, 12]. In a study of children below ninth grade, the overall prevalence of self-injury was 8%, with 9% of girls and 6.7% of boys reporting engagement in self-injury [6]. Girls in ninth grade were also found to engage in self-injury more than three times as frequently as ninth-grade boys [6]. Preadolescent children can have passive or active suicidal ideation, and most reports of self-injury are unknown to caregivers because depressed children and adolescents may exhibit different behavior than depressed adults [12]. As a result, many children at this age who require psychiatric treatment do not receive care, and unfortunately, engaging in self-injury as a child is a precursor to more dangerous psychopathology [2, 12]. Additional studies are required to elucidate the percentage of youth who engage in self-harm behavior that require medical evaluation.

Within the adolescent population, the prevalence of self-injurious behavior does not decrease despite more frequent screening for mental health concerns. In a study of 1802 adolescents, 8% of overall adolescent subjects reported self-harm, with prevalence more common in females (10%) than males (6%) [3]. Adolescents with depression and anxiety who do not receive treatment are more likely to commit self-injury in young adulthood at least once [3]. Furthermore, adolescent self-injurious behavior has been independently associated with antisocial behavior, obesity, alcohol use, cigarette smoking, and chronic cannabis use [3, 13]. Typically, less than half of adolescents with self-injury utilize mental health services [1]. Female patients are more likely to utilize mental health services for depression and self-injury [1]. Of those overall who do seek services, male patients are at increased risk for self-injury, as most utilize services for learning challenges in school due to attention/concentration difficulties or externalizing problems [1]. Additional school support or community resources may help parents and their adolescent children increase family connectedness to improve communication within the family while addressing mental health concerns.

Self-injury in children usually resolves spontaneously in early adulthood, although girls are at higher risk for continued behavior as a young adult compared to boys [4]. Children presenting with self-injurious behaviors should have a thorough psychiatric assessment for other risk factors [1, 3]. Family and other interpersonal support are crucial in implementing treatment recommendations [1, 3, 14]. Treating early symptoms of self-injury and internalization of symptoms prior to adolescent years could be a promising intervention to prevent self-injury or recurrence of destructive behavior. Future studies are required to provide important insights into the sex-specific course of self-injury in children from preadolescence to early adulthood. It is essential for clinicians who interact with youth to recognize that routine health assessment for harmful behaviors may be indicated at an earlier age than previously thought.

## 4. Conclusion

We present the first case of an elementary school-aged child who committed nonfatal self-injury through intentional carbamazepine overdose. Carbamazepine overdose can result in severe central nervous system and cardiac toxicity requiring early identification and rapid interventions to improve patient outcome. Young children who display no obvious symptoms of difficult grief have the cognitive capacity to commit self-injury with intentional ingestion. Intoxication should always be suspected in all children who present to the emergency department with altered mental status to achieve better outcomes. Physicians should educate parents on the importance of supervising their children and being vigilant to keep all medications out of reach. Additional studies should determine psychosocial predictors of self-injury in preadolescent children and understand how to connect those who are at risk with proper mental health services.

## Figures and Tables

**Table 1:** Serial carbamazepine levels during hospitalization.

Carbamazepine level	
Day 0	30.2 *μ*g/mL
Day 1	18.8 *μ*g/mL
Day 2	16.7 *μ*g/mL
Day 3	16.5 *μ*g/mL
Day 4	12.8 *μ*g/mL
Day 5	5.6 *μ*g/mL
Day 6	1.8 *μ*g/mL
Day 7	0.9 *μ*g/mL

## Data Availability

The data used to support the findings of this study are included with the references section of this article.

## References

[1] Steinhoff A., Ribeaud D., Kupferschmid S. (2021). Self-injury from early adolescence to early adulthood: age-related course, recurrence, and service use in males and females from the community. *European Child & Adolescent Psychiatry*.

[2] Nock M. K., Joiner T. E., Gordon K. H., Lloyd-Richardson E., Prinstein M. J. (2006). Nonsuicidal self-injury among adolescents: diagnostic correlates and relation to suicide attempts. *Psychiatry Research*.

[3] Moran P., Coffey C., Romaniuk H. (2012). The natural history of self-harm from adolescence to young adulthood: a population-based cohort study. *Lancet*.

[4] Griffin E., McMahon E., McNicholas F., Corcoran P., Perry I. J., Arensman E. (2018). Increasing rates of self-harm among children, adolescents and young adults: a 10-year national registry study 2007–2016. *Social Psychiatry and Psychiatric Epidemiology*.

[5] Hilt L. M., Nock M. K., Lloyd-Richardson E. E., Prinstein M. J. (2008). Longitudinal study of nonsuicidal self-injury among young adolescents: rates, correlates, and preliminary test of an interpersonal model. *Journal of Early Adolescence*.

[6] Barrocas A. L., Hankin B. L., Young J. F., Abela J. R. (2012). Rates of nonsuicidal self-injury in youth: age, sex, and behavioral methods in a community sample. *Pediatrics*.

[7] Macnab A. J., Birch P., Macready J. (1993). Carbamazepine poisoning in children. *Pediatric Emergency Care*.

[8] Spiller H. A., Carlisle R. D. (2002). Status epilepticus after massive carbamazepine overdose. *Journal of Toxicology, Clinical Toxicology*.

[9] Ozhasenekler A., Gokhan S., Guloglu C., Orak M., Ustundag M. (2012). Benefit of hemodialysis in carbamazepine intoxications with neurological complications. *European Review for Medical and Pharmacological Sciences*.

[10] Costello E. J., Egger H., Angold A. (2005). 10-year research update review: the epidemiology of child and adolescent psychiatric disorders: I. methods and public health burden. *Journal of the American Academy of Child and Adolescent Psychiatry*.

[11] American Psychiatric Association (2013). *Diagnostic and Statistical Manual of Mental Disorders*.

[12] DeVille D. C., Whalen D., Breslin F. J. (2020). Prevalence and family-related factors associated with suicidal ideation, suicide attempts, and self-injury in children aged 9 to 10 years. *JAMA Network Open*.

[13] Kapur N., Cooper J., King-Hele S. (2006). The repetition of suicidal behavior: a multicenter cohort study. *Journal of Clinical Psychiatry*.

[14] Patton G. C., Hemphill S. A., Beyers J. M. (2007). Pubertal stage and deliberate self-harm in adolescents. *Journal of the American Academy of Child and Adolescent Psychiatry*.

